# Electrochemotherapy in the Locoregional Treatment of Metastatic Colorectal Liver Metastases: A Systematic Review

**DOI:** 10.3390/curroncol31110546

**Published:** 2024-11-20

**Authors:** Pierluigi Barbieri, Alessandro Posa, Valentina Lancellotta, David C. Madoff, Alessandro Maresca, Patrizia Cornacchione, Luca Tagliaferri, Roberto Iezzi

**Affiliations:** 1 Department of Diagnostic Imaging and Oncologic Radiotherapy—Emergency and Interventional Radiology Unit, Fondazione Policlinico Universitario “Agostino Gemelli”—IRCCS, 00168 Rome, Italy; pierluigi.barbieri@policlinicogemelli.it (P.B.); alessandromaresca12@gmail.com (A.M.); roberto.iezzi@policlinicogemelli.it (R.I.); 2 Department of Diagnostic Imaging and Oncological Radiotherapy—Gemelli ART (Advanced Radiation Therapy), Interventional Oncology Center (IOC), Fondazione Policlinico Universitario “Agostino Gemelli”—IRCCS, 00168 Rome, Italy; valentina.lancellotta@policlinicogemelli.it (V.L.); patrizia.cornacchione@policlinicogemelli.it (P.C.); luca.tagliaferri@policlinicogemelli.it (L.T.); 3 Department of Radiology and Biomedical Imaging, Yale University School of Medicine, New Haven, CT 06520, USA; david.madoff@yale.edu; 4 Facoltà di Medicina e Chirurgia, Università Cattolica del Sacro Cuore, 00168 Rome, Italy

**Keywords:** electrochemotherapy, colorectal cancer, liver metastases, locoregional therapy

## Abstract

Background: The global incidence of secondary liver cancer is rising due to multiple risk factors, presenting significant challenges in public health. Similarly, colorectal cancer (CRC) remains a leading cause of cancer-related mortality with the development of frequent liver metastases. Surgical resection of CRC liver metastases is only suitable for a limited subset of patients, necessitating alternative nonsurgical treatments such as electrochemotherapy (ECT); Methods: This review adhered to the S.P.I.D.E.R. framework. Systematic searches of PubMed, Cochrane, and Scopus databases were conducted for studies published between 2003 and 2023, following PRISMA guidelines. Inclusion criteria were full-text clinical studies in English focusing on ECT-treated CRC liver metastases, excluding reviews, editorials, and non-clinical papers. The GRADE approach was utilized to assess evidence quality, considering study limitations, consistency, and other factors; Results: From 38 identified articles, 4 met the inclusion criteria, encompassing 78 patients and 128 treated lesions. The studies demonstrated variability in design and follow-up duration (3–11 months). Complete response (CR) rates ranged from 33.3% to 63.0%, while progression disease (PD) rates were high, ranging from 23.0% to 55.6%. Median overall survival (OS) spanned 11.3 to 29.0 months. No severe ECT-related complications were reported. Conclusions: ECT appears to be a safe and effective modality for the treatment of CRC liver metastases, especially for lesions unsuitable for other ablative techniques. Further prospective and randomized studies are essential to better define the role of ECT in managing CRC liver metastases and to compare its efficacy with other ablative methods.

## 1. Introduction

Globally, secondary liver cancer is one of the most common cancers and one of the main leading causes of cancer-related deaths, posing a significant burden on public health [1,2]. Meanwhile, colorectal cancer ranks third in incidence and second in cancer-related deaths globally, with 25–30% of colorectal cancer (CRC) patients developing liver metastases [1]. Despite advancements in oncology and surgery, only a quarter of CRC patients with liver metastases are eligible for resection [3,4]. Local nonsurgical interventions, such as tumor ablation, provide additional treatment options in patients unsuitable for surgical resection. These interventions include thermal techniques like radiofrequency [5,6] or microwave ablation [7] and cryoablation [8], as well as nonthermal approaches like stereotactic body radiotherapy (SBRT) [9], interventional radiotherapy (IRT, also called brachytherapy) [10], irreversible [11], and reversible electroporation (electrochemotherapy, ECT). [12]. The European Society of Medical Oncology (ESMO) recognizes local ablation procedures in the consensus guidelines for metastatic colorectal cancer (mCRC) [13]. Treatment choice depends on various factors, including the characteristics of the target lesion, and must be carefully discussed in multidisciplinary meetings [14]. Thermal ablation techniques are gaining popularity as alternatives to open surgery for both primary and secondary liver tumors, with some limitations such as the size and number of target lesions [14]. In such cases, chemoablation, specifically electrochemotherapy, becomes a valuable addition to local therapies [15]. ECT is based on the reversible electroporation technique, which generates transient permeation of the cell membrane in order to enhance drug delivery to cells, making it effective for various tumors [16,17]. The European Standard Operating Procedures for Electrochemotherapy (ESOPE) has developed guidelines for ECT on cutaneous tumors, and its effectiveness has expanded to deep-seated tumors like those within the liver. Advantages of ECT over other ablative techniques include its ability to be used near critical structures, repeatability, and suitability as a local therapy between chemotherapy cycles [18,19,20]. Literature provides favorable data about patient tolerance to ECT, exhibiting negligible side effects and no significant discomfort, nausea, or systemic repercussions [21,22,23,24]. Therefore, we conducted a systematic review of recent literature to explore the role of ECT in the management of liver metastases of CRC. The aim is to present comprehensive data on clinical outcomes and toxicity associated with percutaneous or intra-surgical ECT of CRC liver metastases.

## 2. Materials and Methods

### 2.1. Formulation of Clinical Questions

Our systematic review adhered to the sample, phenomenon of interest, design, evaluation, research-type (S.P.I.D.E.R) guidelines framework [25], focusing on sample (S), phenomena of interest (PI), design (D), evaluation (E), and research type (R). Specifically, the parameters were colorectal cancer liver metastases (S). Electrochemotherapy (PI), specified qualitative data collection and analysis methods (D), local control (LC), overall survival (OS), toxicity (E), and a qualitative research approach (R).

### 2.2. Outcome Identification

Our study identified two beneficial outcomes, which are local tumor control and overall survival. Local tumor control was defined as complete (CR) or partial response (PR), as shown on computed tomography (CT) or magnetic resonance imaging (MRI) according to Response Evaluation Criteria in Solid Tumors (RECIST) 1.1 criteria [26]. Overall survival was defined as the period starting from the procedure, extending until death occurs, irrespective of its cause. Additionally, harmful outcomes were recognized, encompassing only severe toxicities, which were graded according to the Common Terminology Criteria for Adverse Events (CTCAE v5.0) [27]. All of these outcomes were deemed “critical” for the decision-making process.

### 2.3. Search Methodology and Evidence Selection

By following Preferred Reporting Items for Systematic Reviews and Meta-Analyses (PRISMA) guidelines this systematic review employed systematic searches on PubMed, Cochrane and Scopus to identify comprehensive articles evaluating the efficacy of electrochemotherapy in mCRC with secondary liver lesions [28]. The advanced search utilized medical subject headings (MeSH) and keywords, with the following string: (electrochemotherapies[MeSH Terms]) OR (electrochemotherapies[MeSH Terms]) AND (liver) OR (hepatic) AND (metastases[MeSH Terms]) OR (metastases, neoplasm[MeSH Terms]) OR (neoplasm metastases[MeSH Terms]) OR (metastatic) AND (cancer, colorectal[MeSH Terms]) OR (cancers, colorectal[MeSH Terms]) OR (carcinoma, colorectal[MeSH Terms]) OR (carcinomas, colorectal[MeSH Terms]) OR (colorectal cancer[MeSH Terms]) AND (locoregional therapy) AND (locoregional therapies) to ensure a broad yet sensitive search approach. In addition to MeSH-based searches, a PubMed search without MeSH, employing the keyword “Electrochemotherapy liver metastases”, was conducted. The inclusion criteria encompassed randomized–controlled trials (RCTs), as well as prospective, retrospective, and cohort studies utilizing percutaneous or surgical ECT. The review focused exclusively on full-text clinical studies of mCRC patients treated with ECT alone, excluding conference papers, surveys, letters, editorials, book chapters, and reviews. The search, limited to English-language publications between 2003 and 2023, aimed to ensure relevance within the selected timeframe. Primary endpoints included local tumor control and overall survival, while secondary endpoints were acute and late toxicities (>grade 2). The Grading of Recommendations Assessment, Development and Evaluation (GRADE)pro Guideline Development Tool (GDT) was employed to generate Summary of Findings tables within Cochrane systematic reviews [29]. The evaluation of quality revealed significant clinical data and methodological heterogeneity among the studies included, rendering quantitative synthesis unsuitable. Consequently, the results of meta-analysis were not disclosed. This study has been registered the International Prospective Register of Systematic Reviews (PROSPERO) (ID: CRD42024580330) and adheres to the PRISMA guidelines.

### 2.4. Assessment of Evidence Quality

The level of evidence for all specified outcomes was evaluated using the GRADE approach [28], considering factors such as study limitations, imprecision, indirectness, inconsistency, and publication bias. The level of certainty in the evidence can be categorized as high, moderate, low, or very low.

### 2.5. Benefits/Harms and Clinical Recommendations

Drawing from the evidence summary, considerations were made regarding the balance between benefits and risks associated with the intervention. These judgments were classified as favorable, uncertain/favorable, uncertain, uncertain/unfavorable, and unfavorable. The strength of the recommendation was classified as either strong positive, conditional positive, uncertain, conditional negative, or strong negative.

## 3. Results

**Figure 1:**
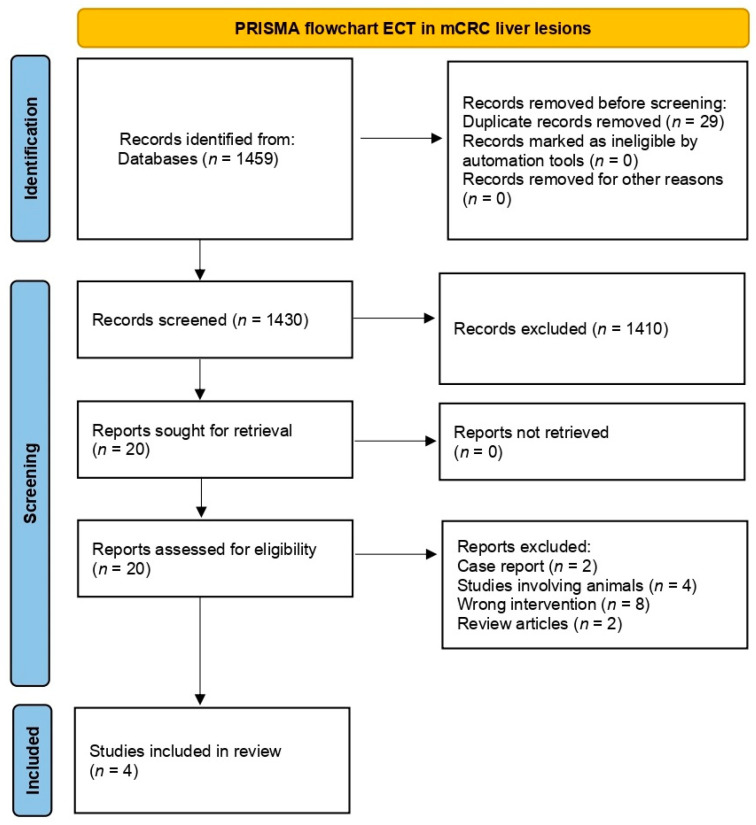
PRISMA flowchart.

Literature search resulted in 1459 articles. Only papers involving liver metastases from CRC were considered. After exclusion of conference papers, surveys, letters, editorials, animal studies, case reports, and reviews, the 20 remaining manuscripts underwent screening by abstract evaluation. After abstract exclusion based on topic, four papers were assessed for eligibility via full-text examination (Figure 1).

Table 1 includes a summary of evidence regarding the four selected studies.

All four articles [30,31,32,33] were included in the final systematic review, totaling 78 patients who underwent ECT for treatment of liver metastases from CRC, for a total of 128 treated lesions. The four selected studies had a prospective observational design; two papers were conceived as a prospective phase I and phase II study. Three studies included patients with CRC liver metastases only, treated with an open-surgery approach [30,31,32], performed by electrodes placement with either variable or fixed geometries, contingent upon the visible metastasis location. One study was performed with only the percutaneous technique [33] by placing up to six probes simultaneously depending on the size, configuration, and location of the target area. Therapy planning was facilitated by software, where the parallel placement of the probes at a specified distance is crucial for effective therapeutic coverage of the target lesion. The objective was to maintain a spacing of 2.0–2.5 cm between probes and apply a voltage of approximately 1000 volts per cm [33]. In the study by Spallek, patients included had multiple histology-confirmed primary neoplasms, in which CRC was the most frequent metastatic liver disease (38.9% of cases, which were the only ones considered for our systematic review) and treated percutaneously [33]. All the papers reported the injection of an i.v. bolus of bleomycin. The same dose was reported in all four articles (15.000 UI/m^2^). In all papers, electric pulses started 8 minutes after the injection under general anesthesia. In Edhemovic’s phase I study [30], they divided patient population (median age 60 years; range 44–69) into the following three groups: In the first one, patients with bilateral, multiple, metachronous metastases underwent a two-stage liver resection due to disease extent or their general condition. In the first operation, the right portal vein was ligated, and left-sided metastases were excised or ablated with radiofrequency ablation. Up to three right-sided metastases were treated with ECT. In the second operation, both treated and untreated right-sided metastases were removed with right partial hepatectomy. In the second group, patients with synchronous metastases, whose general condition and disease extent precluded simultaneous removal of the primary tumor and metastases. The primary tumor was removed in the first operation, and some liver metastases were treated with ECT. About six weeks later, during the second operation, liver metastases were resected. In the third group, patients with up to three metachronous, unresectable liver metastases that were unsuitable for standard thermal ablation due to proximity to major blood vessels, were included. Here, ECT was the only treatment option. The dimension of lesions treated by Edhemovic [30] ranged from 9 to 29 mm according to radiological evaluations. According to the proximity of the metastases to the major blood vessels, they were labeled as either “central” or “peripheral”.

In Edhemovic’s phase II paper (median age 63.1 years; range 35–81) [31], patients who underwent ECT were treated at least with systemic chemotherapy and targeted therapy (bevacizumab or cetuximab) and some of them also received other local treatments (e.g., RT and RFA). Inclusion criteria included at least one unresectable liver metastasis, which either required extensive resection or could not be treated with standard thermal ablative therapies due to its proximity to major blood vessels. The average size of treated lesion was 2 cm (range 0.3–6 cm). The median follow-up was 330 days.

In Coletti’s paper [32], the authors included patients (mean age 64.4 years; SD 13.5) with a histology-confirmed, metachronous or synchronous colorectal liver metastases (CLM) that were not suitable for resection and had been off chemotherapy for more than 30 days prior to ECT. The target tumor lesions for ECT were ≤ 3 cm in size and not previously treated with surgery or locoregional treatments. The lesions also were not resectable due to size, number, location, proximity to vascular/biliary structures, or extent of parenchymal resection and located within 2 cm of the liver capsule. Follow-up was scheduled at 30 days and six months after the procedure.

Spallek and colleagues [33] performed ECT percutaneously under CT-fluoroscopic guidance. Since the paper described a heterogeneous group of secondary lesions, it was not possible to extrapolate specific CRC metastases subgroup characteristics such as median age of population, number and size of lesions, and localization. However, the study demonstrated that ECT was most effective in terms of progression-free survival and overall survival for lesions smaller than 6 cm in diameter compared to those larger than 6 cm (*p* = 0.0209 and *p* = 0.0322, respectively) [33].

Complete response (CR) rates are variable across studies, ranging from 33.3% to 63.0% [30,31,32,33]. One study described an initial CR of 0% (0/9 treated lesions) at 30-day MRI follow-up, increasing to 33% (3/9 lesions) at 6-month MRI follow-up [32]. Progression disease (PD) rates are relatively high, ranging from 23.0% to 55.6% [30,31,32,33]; these data might be hindered by Coletti’s paper, which shows a high PD (5/5 lesion at 6 months follow-up) in a single patient [32]. PR and stable disease (SD) percentages are relatively low.

In terms of OS the median values vary between 12.1 and 29.0 months [31,33]. Coletti reports a 6-month OS and progression-free survival (PFS) rates of 100% and 80%, respectively [32]. However, the availability of OS time without a corresponding ratio (or vice versa) limits the completeness of the analysis. No ECT-related mild or severe complications (>grade 2) were reported.

### 3.1. Evidence-to-Decision (EtD) Framework

Among patients with liver metastases from CRC, ECT showed efficacy in achieving favorable outcomes related to LC and OS. However, the level of certainty associated with these findings is very low.

### 3.2. Harms/Benefits Ratio and Conclusive Recommendation

The results of this systematic review express an uncertain/favorable opinion regarding the balance between benefits and harms according to the GRADE approach. The final recommendation suggests that, for individuals with CRC liver metastases, the consideration of ECT should be approached on a case-by-case basis. This decision should be made at the discretion of the patient and the clinician, following a thorough discussion, preferably within a multidisciplinary team that includes an expert in ECT and percutaneous interventions.

## 4. Discussion

The available data on the involvement of ECT in managing mCRC within the liver exhibits various limitations, including small reported series, retrospective study designs, variations in radiation doses and techniques, limited equipment availability, and differences in institutional expertise and approaches [30,31,32,33]. The contemporary approach to treating cancer patients relies on personalized methods, and integrating various medical disciplines, particularly those involved in locoregional control. By doing this, clinical outcomes can be significantly enhanced, especially in cases of oligometastatic diseases [34]. Although there has been growing interest in this technique, there have been no randomized trials in the literature comparing ECT to other ablative techniques, and very few observational studies involving the use of ECT in liver metastases. ECT was introduced as an alternative to thermal ablation to enhance ablative outcomes through a nonthermal mechanism. Unlike thermal ablation, it operates without causing a heat-sink effect or damage to blood vessels and promotes apoptosis over necrosis. This method relies on a pulsed electrical field rather than Joule heating, facilitating easier physical simulation and more precise intervention planning. ECT employs a locally generated electrical field (electroporation) to temporarily increase the permeability of tumor cell membranes, allowing hydrophilic chemotherapy drugs such as bleomycin to penetrate more effectively. This enhanced permeability reduces the required systemic dosage of the drug while amplifying its local impact. Additionally, electroporation induces vasoconstriction, leading to hypoxic cell injury and prolonged retention of cytostatic drugs within the neoplastic tissue. Unfortunately, the scarcity of robust, useful studies has led to heterogeneous datasets. To address this, we identified and reviewed four articles spanning from 2014 to 2021 consisting of observational prospective single-center experiences. The lack of long-term follow-ups and differently expressed OSs made it challenging to extract relevant data for our patient cohort. Due to these limitations, the traditional P.I.C.O. framework was not applicable. To overcome these challenges, we opted for a qualitative systematic review using the S.P.I.D.E.R. tool. Cooke et al. highlighted the potential value of the S.P.I.D.E.R. search strategy tool, emphasizing its ongoing evolution beyond the Population, Intervention, Comparison, and Outcomes (P.I.C.O) search strategy tool [25]. This approach was deemed suitable for our qualitative systematic review, particularly in the context of qualitative and mixed methods research. The phase I study by Edhemovic and colleagues found that at their first MRI evaluation (median follow-up interval of 33 days from the treatment) CR was present in 23/27 (85%) treated metastases, while PR in 4/27 (15%) lesions showed peripheral contrast at one-month follow-up, indicating residual disease [30]. A subgroup of 14 patients underwent a second evaluation (median follow-up interval of 147 days from the treatment), which showed a CR of 10/14 (71%), whereas 4/14 (28.5%) treated lesions showed progression of the disease (PD) [30]. An interesting histological analysis was conducted by the authors of this study on a subgroup of resected metastases, showing that those lesions not treated by ECT had a significantly higher percentage of residual viable tumor tissue than ECT-treated metastases (*p* ≤ 0.001, two-tailed *t*-test), while there was no difference in terms of response to ECT for metastases located near or far from major blood vessels [30]. This could specifically be attributable to the lack of heat-sink effect, suggesting an interesting application of this method. A subsequent phase II study from the same authors was performed, involving more lesions (84) [31]. The authors found a lower CR at 6 months (53/84, 63%) compared to the previous study’s CR of 85%, and lower PR (10/84, 12%) compared to the previous study’s PR of 15%, with two lesions showing stable disease (SD, 2%) and nineteen lesions showing progression of disease (23%) [31]. The same study also showed a significantly longer median duration of the response of CR metastases (20.8 months) compared to that of PR metastases (*p* < 0.0001). Patients who had a good response to ECT had significantly slower progression of diseases locally or systemically (*p* = 0.0016) than patients with PD. In addition, OS did not differ between responders and non-responders to ECT (*p* = 0.77) [31]. Finally, metastases were equally distributed between central (considered near major vessels; 44%) and peripheral (56%) locations showing no significant difference in the local metastasis control rate between the two sites (*p* = 0.22) [31]. In a small population, Coletti and colleagues showed a CR of only 33.3% lesions (4/9) at 6 months, with a progression of disease of 55.5% (5/9). However, the five metastases undergoing progression of diseases all came from a single patient with a complicated postoperative systemic infection and a subsequent delay in adjuvant chemotherapy [32]. All patients reached a 6-month OS, and four out of five patients (80%) had PFS of 6 months [32]. Interestingly, Spallek and colleagues achieved local control in 6/8 CRC lesions, with 4/8 CR (50%) and 2/8 PR (25%) [33]. In addition, their manuscript underlined ECT’s best performance (PFS and OS) in lesions within 3 and 6 cm diameters (*p* = 0.0242 and = 0.0297, respectively) [33].

Despite the aforementioned limitations, this systematic review shows that ECT is efficient and safe, and it could be considered as a valid treatment option in locoregional control of lesions that exceed the thermal ablation capacity, tumors insensitive to radiation, or lesions situated near organs vulnerable to radiation or temperature changes. ECT seems particularly well-suited for treating centrally located liver metastases, especially in proximity to or infiltrating major vessels (Vp3-Vp4 portal vein thrombosis), as these lesions are not amenable to resection and are unsuitable for radiofrequency or microwave ablation due to the heat-sink effect. In comparison with the paper by Granata et al. [15], our systematic review focuses only on CRC application of ECT, combining data regarding local tumor control (CR and PR), and the overall survival (OS) of the four aforementioned studies, providing a recommendation on the use balanced between harms and benefits.

## 5. Conclusions

This systematic review indicates that ECT might be effective in treatment of colorectal liver metastases with a solid security profile. It would be beneficial to conduct more prospective or randomized studies comparing ECT and other ablative techniques in colorectal metastatic disease setting. This approach aims to enhance the quality of evidence and facilitate more informed decisions regarding the appropriate treatment choice.

## Figures and Tables

**Table 1:** Summary of evidence.

Autors	Period	Study	Patients	Treatment	LC	PFS	OS	Toxicity	FU (Months)	Main Results
**Edhemovic [30]**	2009–2012	Prospective Phase I	16 patients, 27 CRC lesions	**Surgical**	At 33 days (range 14–76): -CR 23 (85%) -PR 4 (15%) At 147 days (range 31–274) -CR 10 (71%)			No major complications		-Metastases not treated by ECT had a significantly higher percentage of residual vital tumor tissue compared to ECT-treated metastases -No difference in response of metastases located near or distant to major blood vessels
**Edhemovic [31]**	2011–2018	Prospective Phase II	39 patients, 84 CRC lesions	**Surgical**	At 330 days: -(CR) 53 (63.0%) -(PR) 10 (12.0%) -(SD) 2 (2.0%) -(PD) 19 (23.0%)		29.0 months	No major complications	Mean: 330 days (11 months)	-Median duration of CR was 20.8 months, significantly longer than the PR (*p* < 0.0001) -No significant differences (*p* = 0.22) in terms of response to lesion location -Patients with good response to ECT had significantly slower local or systemic disease progression (*p* = 0.0016) than patients with PD -No differences in OS between responders and non-responders to ECT (*p* = 0.77)
**Coletti [32]**	2017	Prospective	5 Patients, 9 CRC lesions	**Surgical**	At 30 days: -CR 0 -PR 5 (55.5%) -SD 4 (45.5%) At 6 months: -CR 3 (33.3%) -SD 1 (11.1%) -PD 5 (55.5%)	(Rate) 80% at 6 months	(Rate) 100% at 6 months	No major complications	6 months	-All patients reached a 6-month OS -4 out of 5 patients had a 6-month progression-free survival
**Spallek [33]**	2018–2020	Prospective	18 patients, 8 CRC lesions	**Percutaneous**	At 3 months: -CR 4 (50%) -PR 2 (25%) -NE 2 (25%)	7.3 ± 12.1 months	12.1 ± 12.1 months	No major complications	Mean: 9 months	-ECT performed best (PFS and OS) in lesions between 3 and 6 cm in diameter (*p* = 0.0242; *p* = 0.0297) -ECT effectiveness was not dependent on the lesion location -PFS and OS were not dependent on the tumor histology

Table legend: CRC: colorectal cancer; LC: local control; CR: complete response; PR: partial response; SD: stable disease; PD: progressive disease; PFS: progression-free survival; OS: overall survival; FU: follow-up; ECT: electrochemotherapy.
